# Increased proinflammatory cytokines in tears correspond with conjunctival SARS-CoV-2 positivity in symptomatic COVID-19 patients

**DOI:** 10.1038/s41598-022-11285-7

**Published:** 2022-05-04

**Authors:** Anna Niedźwiedź, Miłosz Kawa, Ewa Pius-Sadowska, Agnieszka Kuligowska, Alicja Ziontkowska, Dawid Wrzałek, Marta P. Wiącek, Miłosz Parczewski, Andrzej Ossowski, Grażyna Zielińska, Krzysztof Safranow, Krzysztof Kozłowski, Bogusław Machaliński, Anna Machalińska

**Affiliations:** 1 Department of General Pathology, Pomeranian Medical University in Szczecin, Al. Powstańców Wielkopolskich 72, 70-111 Szczecin, Poland; 2 First Department of Ophthalmology, Pomeranian Medical University, Al. Powstańców Wielkopolskich 72, 70-111 Szczecin, Poland; 3 Department of Infectious, Tropical Diseases and Immune Deficiency, Pomeranian Medical University in Szczecin, Arkońska 4 Street, 71-455 Szczecin, Poland; 4 Department of Forensic Genetics, Pomeranian Medical University in Szczecin, Al. Powstańców Wielkopolskich 72, 70-111 Szczecin, Poland; 5 Department of Biochemistry and Medical Chemistry, Pomeranian Medical University, Al. Powstańców Wielkopolskich 72, 70-111 Szczecin, Poland; 6 Department of Constitutional Law, Faculty of Law and Administration of the Jagiellonian University in Krakow, Bracka 12 Street, 31-005 Kraków, Poland

**Keywords:** Viral infection, Acute inflammation

## Abstract

Tear fluid cytokine levels may serve as biomarkers of innate immune system response against SARS-CoV-2 infection. Therefore, our aim was to analyze panel of selected inflammatory cytokines in tears of COVID-19 patients in relation to presence of SARS-CoV-2 viral load in conjunctival secretions. In this study concentrations of TNF-α, IL-1b, IL-2, IL-4, IL-5, IL-6, IL-8, IL-10, IL-12 p70, GM-CSF, and IFN-γ were determined by a magnetic bead assay in tear film collected from 232 symptomatic COVID-19 patients. SARS-CoV-2 ocular infection was confirmed based on positive conjunctival swab-based RT-PCR testing. Viral RNA in conjunctival sac was detected in 21 patients (9%). No relation between presence and the duration of ophthalmic symptoms and SARS-CoV-2 infection detected in conjunctival secretions was found. The tear film concentrations of IFN-γ, TNF-α, IL-5, IL-8 and GM-CSF were found to be significantly greater among patients with positive conjunctival swab results as compared to the group negative for SARS-CoV-2 in conjunctival sac. Our current data depict a group of inflammatory mediators in human tears, which may play a significant role in ocular pathology of SARS-CoV-2 conjunctival infection.

## Introduction

Severe acute respiratory syndrome coronavirus 2 (SARS-CoV-2) was first detected in December 2019 in Wuhan, China, and quickly spread worldwide. Coronavirus disease (COVID-19) is a highly contagious disease whose progression can lead to acute respiratory distress syndrome or even death. The virus is primarily spread between people during close contact, often via small droplets produced by coughing, sneezing or talking. People may also become infected by contact with contaminated surfaces and then touch their faces. Although the majority of COVID-19 cases are asymptomatic and mild, with the most common signs being cough, fatigue, fever, muscle pain and dyspnea, some patients develop severe SARS-CoV-2 infection, which can rapidly affect the pulmonary and cardiovascular systems as well as endocrine glands and kidney function^1^. There is also evidence of neurological disturbances and impaired function of special senses with the presence of olfactory, gustatory and auditory disorders that encompass the broad spectrum of clinical manifestations, including anosmia and dysosmia, ageusia and dysgeusia, or ear hypoacusis^2^.

The coronavirus family has previously been associated with conjunctivitis, which produces the “red eye” symptom in humans. It is believed that vasculitis is an important underlying contributor to the ocular manifestations seen following coronavirus eye infections, which include conjunctivitis, anterior uveitis, retinal vasculitis and choroiditis. Conjunctivitis, although rare, could also be the first COVID-19 presentation^3^. Recently, it was suggested that unprotected exposure of the eyes to SARS-CoV-2 serves as a viral entry portal to the entire organism. Eye care professionals are in close proximity to patients during various ophthalmic procedures, including basic ophthalmic examination, and therefore may present a relatively increased risk of contracting SARS-CoV-2. Indeed, one of the first health care professionals, who alerted the world about first symptoms of COVID-19 among people, was the Chinese ophthalmologist Li Wenliang, who died from COVID-19 on February 7, 2020, at the age of 34, after he had caught SARS-CoV-2 from an asymptomatic COVID-19 patient with glaucoma who was treated at the ophthalmology ward in the town hospital of Wuhan^4^.

The fact that the ocular manifestation can be the first presenting feature of novel coronavirus disease should not be ignored, and the possibility of spreading SARS-CoV-2 through ocular secretions cannot be ruled out. Colavita et al. analyzed the case of one of the first European COVID-19 patients who travelled from Wuhan to Italy and presented symptoms of dry cough, sore throat and bilateral conjunctivitis. Viral infection was confirmed by ocular swab detection, demonstrating the presence of SARS-CoV-2 in the tear sample^5^. Whether the eyes are one of the preliminary sites of virus transmission from the environment to the lungs via the lacrimal passage or the virus gets to the eyes in retrograde mode through the airway passages via the lacrimal system to the eyes is being further examined. In addition, the local expression of angiotensin-converting enzyme 2 (ACE-2) receptors on the ocular surface and their cellular density are still under study. The conjunctiva is directly exposed to extraocular pathogens, and the mucosa of the ocular surface and upper respiratory tract is connected by the nasolacrimal duct and may share some common entry receptors for various respiratory viruses, such as SARS-CoV-2^6^.

Ocular manifestation of COVID-19 is a hot topic given that the eye allows for direct inspection of the inflammatory changes in conjunctival structures without biopsy^7,8^. Likewise, tears are a convenient, noninvasive source in which biomarkers can be analyzed. As cytokines play a significant role as essential mediators of the inflammatory response during a viral infection, they have also been recently tested in tears of COVID-19 patients and compared to healthy controls, indicating that out of twenty-seven cytokines that were assessed, the increase was observed in 7 of them (i.e., IL-9, IL-15, G-CSF, GM-CSF, IFN-γ, PDGF and VEGF) along with a decrease in eotaxin concentration^9^. However, tear cytokines were not correlated with the viral load in conjunctival secretions in that study.

Hence, we conducted a study to check whether there is a risk of isolated conjunctival viral activity in a large number of symptomatic COVID-19 patients with positive nasopharyngeal swab-based RT–PCR results. Furthermore, SARS-CoV-2 infection detected in conjunctival secretions of COVID-19 patients was then evaluated as a potential cytokine stimulant of local inflammatory reactions, thus corroborating the hypothesis that the conjunctival sac is a potential site of ocular involvement in active SARS-CoV-2 infection.

## Results

### Clinical data of patients with COVID-19

A total of 232 SARS-CoV-2-positive symptomatic patients (104 females and 128 males) were included in the study. Of these, 21 patients (9%) had positive conjunctival swab results. The characteristics of patients with positive and negative SARS-CoV-2 conjunctival swab results are shown in Table 1. Accordingly, there were no differences between the groups in age, sex, BMI, and smoking status of the patient or current medication use between groups, with one exception: slightly lower NSAID use among patients with negative conjunctival swab results than among those with positive results was observed (11.37% patients with negative conjunctival swab results vs. 28.57% of patients with positive conjunctival swab results; p = 0.04). No significant relationship was found between virus detection in the conjunctival sac and comorbidities such as hypertension, diabetes, ischemic heart disease, hypercholesterolemia, liver, respiratory tract and different rheumatic diseases or cancer at COVID-19 diagnosis. Neither the stage of disease at the time of hospital admission nor the presence of pneumonia, fever above 38 °C, dyspnea, chest pain, cough, smell/taste abnormalities, headache or diarrhea were associated with the presence of the virus on the ocular surface (Table 2). Similarly, the implemented treatment was also not specifically related to the expression of virus in the conjunctival sac, e.g., respirator-assisted therapy in the intensive care unit (2.37% patients with negative conjunctival swab result vs. 0% patients with positive conjunctival swab result; p = 1.00), hemodialysis (2.84% patients with negative conjunctival swab result vs. 9.52% patients with positive conjunctival swab result; p = 0.16) or oxygen therapy (49.76% patients with negative conjunctival swab result vs. 61.90% patients with positive conjunctival swab result; p = 0.36).

**Table 1:** General characteristics of patients with positive and negative conjunctival swab results.

Parameter	Patients with negative conjunctival swab results	Patients with positive conjunctival swab results	*p*
Age (mean ± SD)	55.27 ± 15.09	58.29 ± 14.28	0.47
Sex (male/female)	112/99	16/5	0.06
Body-mass index (mean ± SD)	28.64 ± 5.42	28.78 ± 4.74	0.89
**% of patients with a given parameter**			
Medical history			
Hypertension	39.34	47.62	0.49
Diabetes	17.06	23.81	0.55
Ischemic heart disease	6.16	9.52	0.63
Hypercholesterolemia	12.32	14.29	0.73
Liver disease	0.95	0.00	1.00
Respiratory tract disease	9.00	14.29	0.43
Rheumatic disease	8.53	0.00	0.38
Cancer	9.95	19.05	0.26
Other diseases	33.65	38.10	0.81
Tobacco use	40.28	42.86	0.82
Currently taken medications			
Drugs taken on permanent basis	64.45	61.90	0.81
Statins	15.64	9.52	0.75
NSAIDs	11.37	28.57	**0.04**
Antihypertensive drugs	40.76	52.38	0.36
Anticoagulants	6.16	14.29	0.16
Cardiac drugs	6.64	9.52	0.64
Anti-asthmatic drugs	9.00	4.76	1.00
Other drugs	39.81	42.86	0.81

Statistically significant p values are shown in bold.

**Table 2:** Data regarding disease severity and the presence and duration of COVID-19 symptoms in patients with positive and negative conjunctival swab results.

Parameter	Patients with negative conjunctival swab result	Patients with positive conjunctival swab result	*p*
Disease severity at the hospital admission (median (IQR))	2 (1)	2 (0)	0.49
**% of patients with a given parameter**			
Fever above 38 °C	63.03	66.67	0.82
Dyspnea	39.81	57.14	0.16
Cough	72.51	66.67	0.61
Chest pain	24.17	28.57	0.61
Smell/taste abnormalities	40.76	33.33	0.64
Headache	39.34	23.81	0.24
Diarrhea	25.59	28.57	0.79
Pneumonia	87.44	85.71	0.74
**Duration—days (mean ± SD)**			
Fever above 38 °C	6.23 ± 3.26	5.93 ± 3.29	0.91
Dyspnea	6.61 ± 3.68	6.33 ± 3.50	0.98
Cough	6.71 ± 4.04	6.14 ± 3.35	0.80
Chest pain	6.51 ± 3.81	7.67 ± 3.33	0.37
Smell/taste disorders	5.85 ± 4.06	7.57 ± 2.88	0.13
Headache	6.07 ± 3.49	6.20 ± 3.70	0.96
Diarrhea	4.54 ± 2.98	7.00 ± 3.41	0.06

### Ophthalmological data of patients with COVID-19

Then, we analyzed the presence and duration of ophthalmic symptoms among the patients with positive and negative conjunctival swab results. Among patients with negative conjunctival swab results, 15.35% had at least 1 ECS at the time of examination in the admission department. At the same time, 19.05% of patients with the presence of the virus on the ocular surface had at least 1 ECS (p = 0.75). Accordingly, we found no significant differences in the presence of ocular symptoms within 7 days preceding the examination at the emergency unit (20.30% of patients with negative conjunctival swab results vs. 28.57% of patients with positive conjunctival swab results; p = 0.40). When analyzing the cumulative ECS, no differences between groups were observed both at the time of examination (p = 0.67) and within 7 days preceding admission to the emergency department (p = 0.34).

In addition, to evaluate the potential impact of concomitant ocular diseases, medication use and past ocular surgeries on SARS-CoV-2 infection in the conjunctival sac, we analyzed the data regarding current ophthalmic history between the groups. We found no differences between the groups in the presence of allergic conjunctivitis (2.97% patients with negative conjunctival swab results vs. 9.52% patients with positive conjunctival swab results; p = 0.17), dry eye (4.95% patients with negative conjunctival swab results vs. 4.76% patients with positive conjunctival swab results; p = 1.00) or infectious conjunctivitis (2.97% patients with negative conjunctival swab results vs. 4.76% patients with positive conjunctival swab results; p = 0.50) within one year preceding the COVID-19 event. Similarly, we found no differences between the groups in regard to contact lens wear (4.46% patients with negative conjunctival swab results vs. 4.76% patients with positive conjunctival swab results; p = 1.00), past ocular injuries (0.99% patients with negative conjunctival swab results vs. 0% patients with positive conjunctival swab results; p = 1.00), having undergone ocular surgeries (2.97% patients with negative conjunctival swab results vs. 4.76% patients with positive conjunctival swab results; p = 0.50), or the therapeutic use of drops in dry eye syndrome (6.93% patients with negative conjunctival swab results vs. 9.52% patients with positive conjunctival swab results; p = 0.65).

### Levels of selected cytokines in tears of patients with COVID-19

**Figure 1:**
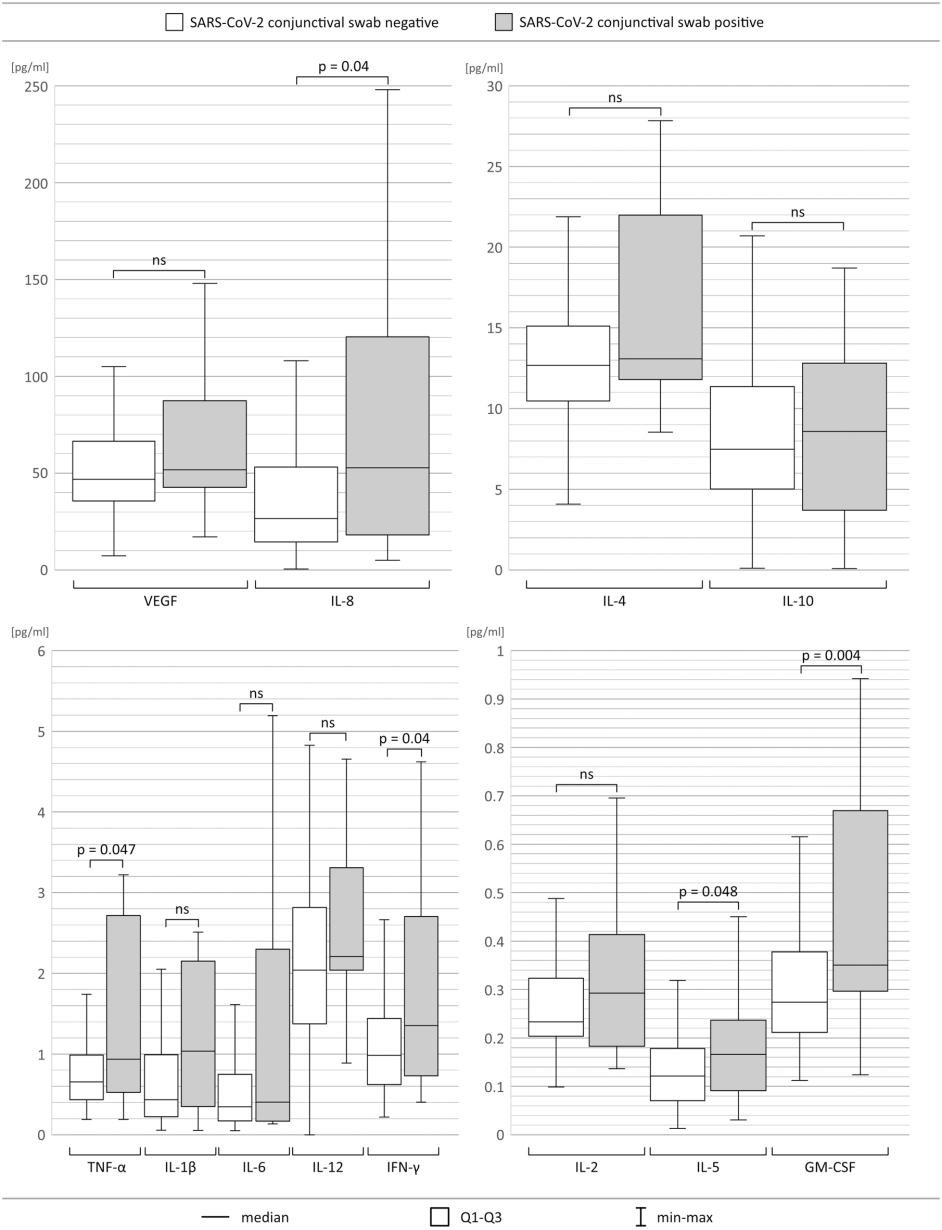
Boxplots showing tear film cytokine levels in patients with SARS-CoV-2-positive and SARS-CoV-2-negative conjunctival swab results. Statistically significant p values are written above the boxplots. *ns* non statistic.

Then, we aimed to investigate the concentrations of selected cytokines in the collected tear film samples from enrolled subjects suffering from COVID-19. The tear film concentrations of IFN-γ, TNF-α, IL-5, IL-8 and GM-CSF were found to be significantly greater among patients with positive conjunctival swab results than among the group that was negative for SARS-CoV-2 in the conjunctival sac (Fig. 1 and Table 3). Interestingly, patients who required hemodialysis were found to demonstrate increased IFN-γ, TNF-α, IL-1β, IL-2, IL-6, IL-8, IL-12, GM-CSF and VEGF concentrations in their analyzed tear film. Additionally, in the case of IL-1β, its tear film level correlated positively with the stage of the disease according to the PAoEaI classification (Rs =  + 0.15; p = 0.035). This may indicate that patients with greater COVID-19 severity had higher levels of such proinflammatory cytokines. Accordingly, the IL-1β tear film concentration was found to be higher in patients with pneumonia complicating COVID-19 (median [IQR] = 0.52 [0.96] pg/ml for patients with pneumonia vs 0.28 [0.29] pg/ml for patients without pneumonia; p = 0.0041).

**Table 3:** Tear film cytokine levels among patients with positive and negative conjunctival swab results (pg/mL).

Parameter	Patients with negative conjunctival swab results	Patients with positive conjunctival swab results	*p*
	Mean ± SD	Mean ± SD	
TNF-α	1.18 ± 2.67	2.05 ± 2.66	**0.047**
VEGF	53.24 ± 28.04	65.96 ± 36.41	0.11
IL-2	0.30 ± 0.21	0.35 ± 0.24	0.40
IL-1β	2.04 ± 8.37	3.39 ± 6.82	0.08
IL-4	13.59 ± 5.10	16.32 ± 6.15	0.11
IL-5	0.15 ± 0.15	0.21 ± 0.17	**0.048**
IL-6	1.15 ± 4.63	1.30 ± 1.59	0.25
IL-8	82.72 ± 301.01	135.32 ± 253.02	**0.04**
IL-10	8.66 ± 5.77	8.40 ± 6.11	0.84
IL-12	2.29 ± 1.59	2.61 ± 0.99	0.06
GM-CSF	0.37 ± 0.45	0.50 ± 0.37	**0.004**
IFN-γ	1.17 ± 0.77	1.72 ± 1.23	**0.04**

Statistically significant p values are shown in bold.

## Discussion

In this study, we investigated the prevalence of SARS-CoV-2 on the ocular surface and elucidated an exceptional profile of several selected cytokines in the tear film of patients with positive conjunctival swab results for SARS-CoV-2 infection. We found that 9% out of 232 symptomatic COVID-19-positive patients had positive results for SARS-CoV-2 conjunctival secretions. The outcomes of this study confirmed the data reported in the other trials. The preliminary data on the presence of SARS-CoV-2 RNA in the conjunctival sac were published online on March 31, 2020, by Wu et al., who examined the conjunctiva in a small series of 38 patients with presumed COVID-19 who were hospitalized in Hubei Province in China during the first few months after epidemic onset. Out of 28 patients who were positive for COVID-19 on RT–PCR from nasopharyngeal swabs, only 2 patients (5.2%) yielded positive findings for SARS-CoV-2 in their conjunctival and nasopharyngeal specimens. Of note, no other specific ocular parameters were investigated^10^. Likewise, similar to our rate, 7.5% positive RT–PCR results from conjunctival swabs in patients with confirmed SARS-CoV-2 infection were reported by Atum et al.^11^. In another small study testing the conjunctival secretions and tears (collected twice over 2–3 days) of 30 confirmed COVID-19 patients, the presence of viral RNA (in both samples) was demonstrated in only one patient, who also showed clinical signs of conjunctivitis^12^. A systematic meta-analysis based on a review of the published literature estimated the general rate of SARS-CoV-2 viral shedding in tears and conjunctival secretions tested by RT–PCR to be 2.7% (11 out of 414 patients examined in 12 different reviewed trials)^13^. As the conflicting results of conjunctival carriage of SARS-CoV-2 in the current literature may be due to variabilities in sampling technique and sampling time window, the results should be interpreted with caution. In particular, the time when samples were obtained from patients may vary between the studies selected for the meta-analysis. Supposedly, samples obtained late during disease development could not yield positive results due to diminishing virus concentrations and specific neutralizing antibody production against SARS-CoV-2. Additionally, the severity of COVID-19 disease might affect the final results of SARS-CoV-2 viral load detection in specific tissues. To the best of our knowledge, this is the first study in the literature that analyses the SARS-CoV-2 viral load in conjunctival secretions collected from a much greater number of symptomatic COVID-19 patients than in other published studies. Moreover, we collected all the samples exclusively from symptomatic patients at the moment of hospital admission due to the COVID-19 diagnosis, but before commencement of any specific antiviral treatment, to exclude potential bias that may lead to false-negative results during the study. Important observations were recently reported by Colavita et al., who found that SARS-CoV-2 may continue to replicate in the conjunctival sac even after the complete clinical recovery of the patient^5^. Another factor that also deserves attention, while investigating the ocular surface as a possible transmission route for SARS-CoV-2, is the lower ACE2 receptor expression on the cornea and conjunctival epithelia in relation to other tissues, such as the lung and heart^14^. Thus, by lowering the requirements necessary for viral invasion, the ocular surface may further become a less-preferred route for this coronavirus. Moreover, a recent study evaluating the replication of actual SARS-CoV-2 and previous SARS-CoV-1 in ex vivo human conjunctival cells showed that both viruses demonstrated a positive replication mechanism, with SARS-CoV-2 revealing even more extensive infectivity than SARS-CoV-1^15^. This again further emphasizes the anterior eye segment as a potential entry site for coronaviruses.

To obtain more indisputable proof of the presence of active SARS-CoV-2 infection in the conjunctival sac of patients with COVID-19, we also detected several proteins in tears, e.g., selected cytokines and growth factors. Indeed, COVID-19 patients present a characteristic cytokine profile in the blood, which has been investigated by many groups. For example, Chi et al. reported higher blood levels of interleukin (IL)-1β, IL-1RA (IL-1 receptor antagonist), IL-2, IL-2Rα, IL-6, IL-7, IL-8, IL-9, IL-10, IL-13, IL-15, IL-17, IL-18, fibroblast growth factor basic (FGF basic), granulocyte colony-stimulating factor (G-CSF), granulocyte-monocyte colony-stimulating factor (GM-CSF), interferon (IFN)-γ, interferon gamma-induced protein (IP10), IFN-α2, monocyte chemoattractant protein-1 (MCP-1), macrophage inflammatory protein (MIP)-1A, MIP-1B, platelet-derived growth factor (PDGF), tumor necrosis factor (TNF)-α, and vascular endothelial growth factor (VEGF) in symptomatic patients with SARS-CoV-2 than in healthy adults^16^. In addition to those found by Chi et al., Hu et al. also detected higher serum levels of similar cytokines in COVID-19 patients than in recruited controls^17^. Moreover, in SARS-CoV-2–infected individuals, proinflammatory cytokines, such IL-6, IL-10, and TNF-α, surge during illness and decline during recovery^18^, indicating that their expression is directly dependent on active viral infection. Importantly, we found here that the tear film concentrations of IFN-γ, TNF-α, IL-5, IL-8, and GM-CSF were significantly greater among patients with positive conjunctival swab results for viral load than among patients with negative conjunctival swab results.

The importance of IFN-γ for the local immune system of the eye stems in part from its ability to clear virally infected cells through the promotion of cytotoxic T-cell responses. More importantly, due to its immunostimulatory and immunomodulatory effects, IFN-γ is responsible for the activation of migrating macrophages to produce a wide array of inflammatory mediators, including TNF-α, which may be released locally to the conjunctival sac fluid as part of the IFN-γ-directed innate immune response^19^. In our study, both TNF-α and IFN-γ, which are critical for innate and adaptive immunity against viral infections, were found to be significantly elevated in tears from COVID-19 patients with active viral infection. Elevated levels of TNF-α and its receptors were also observed in animal models of ocular infections with other coronavirus types, in which this virus was intravitreally inoculated in mouse eyes to study their effect on ocular degeneration^20^. This observation further supports the hypothesis of direct immune reactivity during SARS-CoV-2 infection of the conjunctival sac, observed in our study, in the group with positive conjunctival swab results. Both inflammatory and structural cells of the eye, such as epithelial cells and fibroblasts, act as local sources of cytokines^21^. In particular, exposure of epithelial cells and keratocytes to TNF-a was found to stimulate IL-8 synthesis in these cells^22^. The significant TNF-a release in the anterior segment of the eye during the inflammatory state in the course of COVID-19 may also affect the local corneal epithelial cells, stimulating them to produce IL-8, which is in line with the higher concentration of this cytokine in the conjunctival sac of patients with positive SARS-CoV-2 results. Colony-stimulating factor for granulocytes and monocytes (GM-CSF) stimulates the proliferation of white blood cells, directly contributing to the maintenance of innate immune homeostasis. GM-CSF can also enhance proinflammatory cytokine release and CD4+ T cell differentiation^23^. Recent evidence indicates that pathogenic Th1 cells, with both IFN-γ and GM-CSF high expression, have been found in critically ill COVID-19 patients^24^. Our results of tear analysis from COVID-19 patients may further support this direct role of GM-CSF in SARS-CoV-2 infection.

Notably, only a few studies have investigated conjunctival immune responses to virus infection. Epidemic keratoconjunctivitis (EKC) is a highly contagious and severe viral conjunctivitis that occurs due to human adenovirus infection^25^. It has been documented that interleukin-8 may play a role in the development of subepithelial infiltrates in adenovirus keratitis^26^. This observation goes in line with our study since we have showed increased level of IL-8 in conjunctival secretions from patients with SARS-CoV-2-positive conjunctival swabs. On the other hand, IL-1β, IL-6, and IL-8 were significantly elevated in tears from bacteria affected eyes^27^. This indicate that different pathogens may stimulate specific pattern recognition receptors on cellular surface leading to distinct immune responses. Interestingly, it was also found that allergic inflammation in seasonal and chronic allergic conjunctival disease is associated with increased levels of a number of key cytokines in tears, e.g. IL-2, IL-4, IL-5, IL-10, TNF-α and IFN-γ^28,29^. The role of IL-4, IL-5, IL-10, IFN-γ and TNF-α is also highlighted in pathogenesis of vernal and atopic keratoconjunctivitis^30,31^. Altogether, in different types of conjunctivitis (i.e. viral, bacterial or allergic) diverse pathogenic factors are capable of inducing and orchestrating the secretion of several specific immune factors, which in great part are uniform, and mostly are responsible for similar symptoms in affected eyes. Thus, tear film cytokine profile in course of viral SARS-CoV-2 infection may present numerous similarities with cytokine profiles in other inflammation-based conjunctival disorders.

Our actual results and data from other recent studies have shown that the ocular manifestations in infected humans with symptomatic COVID-19 are generally mild, which may implicate low ocular tropism of SARS-CoV-2, including ocular anterior compartment structures, such as the conjunctival sac. Importantly, we found no relationship between the presence and duration of ophthalmic symptoms and SARS-CoV-2 infection detected in conjunctival secretions. These data are in line with the results of previous studies. In a recently published meta-analysis by Sarma et al. (2020), it was shown that 3.17% of COVID-19 patients had conjunctivitis/red eye; however, only 0.70% of patients had conjunctivitis as the first symptom of the disease, and only 1.94% patients were positive for SARS-CoV-2 by genetic testing in tear or conjunctival samples. Despite the presence of the virus in the tears, only 33.3% showed signs of conjunctivitis or conjunctival chemosis or red eye^32^. Similarly, Wu and associates reported positive conjunctival RT–PCR results in only two out of 12 patients with ocular symptoms and positive SARS-CoV-2 nasopharyngeal swabs^10^. In another attempt to study the potential ocular transmission of COVID-19, Zhou et al. retrospectively tested the conjunctival swabs of 63 patients with confirmed COVID-19 by PCR and found that only three cases with no ocular symptoms were positive, while one patient presenting with symptoms of mild conjunctivitis alone in the same study, who was an anesthesiologist who acquired the infection from a confirmed COVID-19 patient during intubation, had a negative conjunctival swab result^33^. Thus, the absence of ocular symptoms does not mean that the virus is not present in the tear film, and conversely, the appearance of ocular symptoms is not an indication of the presence of the virus in tears. However, in our study, we took it one step further and showed that positive viral detection in conjunctival secretions was significantly correlated with increased concentrations of various inflammatory cytokines in tears, thus indicating that active SARS-CoV-2 infection of conjunctival tissues is able to induce a strong innate immune response against pathogens invading the anterior segment of the eye. Importantly, regardless of these discrepancies, health personnel should take all the necessary precautionary measures regardless of whether patients have ocular symptoms since negative results may not reflect a true absence of the virus in a patient’s conjunctival mucosa. Interestingly, we found no significant correlation between the presence of virus in the conjunctival sac and the presence and duration of COVID-19 symptoms such as pneumonia, high fever, dyspnea, cough, chest pain, smell/taste abnormalities, headache or diarrhea. Indeed, tears have a wide variety of antimicrobial peptides and immunoglobulin that block the possibility of establishing a productive infection in the ocular mucosa, regardless of the stage of viral infection in the rest of the human body. There is great heterogeneity of sialylated ocular mucins and secretory peptides synthesized and secreted by different regions and cell types within the human ocular surface and nasolacrimal ducts that create unique antimicrobial host defense against pathogens such as coronaviruses^34^.

In conclusion, the activation of ocular immune responses observed in the group of symptomatic COVID-19 patients with positive conjunctival swab results may indicate the presence of active infection with SARS-CoV-2 and might carry some important therapeutic implications. Although the percentage of patients who tested positive for SARS-CoV-2 in the ocular fluid was quite low, this group had significantly increased concentrations of several inflammation-related cytokines. Diverse cytokine profiles detected by our group corroborate the inflammatory nature of SARS-CoV-2 infection. In particular, high levels of INF-gamma in tears accompanied by viral load in the conjunctival sac indicate a host reaction against an eye-invading pathogen. Further molecular studies are needed to confirm or refute these results. In particular, more studies are warranted to detect the existence of SARS-CoV-2 in different ocular tissues to clarify the existing ambiguities.

## Materials and methods

### Study group

This retrospective cohort study included 232 patients diagnosed with COVID-19 in the Department of Infectious, Tropical Diseases and Immune Deficiency of Pomeranian Medical University in Szczecin, Poland, based on positive RT-polymerase chain reaction (RT–PCR) results for SARS-CoV-2 obtained from nasopharyngeal swabs. All patients enrolled in the study underwent tear sampling and collection of conjunctival swabs for SARS-CoV-2 detection performed less than 24 h after admission to the emergency unit. All participants underwent a complete general health examination and completed a detailed questionnaire regarding the most common chronic diseases and related medications taken. Participants were also asked to complete a detailed ophthalmologic health questionnaire. An informed consent form in accordance with the tenets of the Declaration of Helsinki was signed by all patients before study enrolment.

### General medical assessment

All patients were interviewed and examined to collect information on the presence of signs and symptoms such as fever, dyspnea, cough, common cold, sore throat, fatigue, chest pain, smell/taste abnormalities, headache and body aches or diarrhea. Patients were also interviewed for anamnesis, the severity and duration of the abovementioned symptoms, the presence of coexisting conditions, and recent exposure history, including travel history and close contact with other known COVID-19 patients. Information regarding the laboratory findings, need for oxygen or respirator-assisted therapy, presence of pneumonia in chest computed tomography (CT) scans, need for hemodialysis or whether the patient died was collected from electronic medical records. The demographic details, family history of different diseases and other general health risk factors present at the time of the enrolment, such as hypertension, hyperlipidemia, tobacco use, diabetes mellitus, cardiovascular, liver, respiratory and rheumatic diseases and prior cerebrovascular events, were recorded. All patients underwent internal examination focused on the respiratory and cardiovascular systems. The severity of the disease was assessed at the moment of hospital admission based on the classification given at the official guidelines of the Polish Association of Epidemiologists and Infectiologists (PAoEaI)^35,36^. The final assessment, as recommended by the PAoEaI, included the duration of the disease, oxygen saturation measured with the use of pulse oximetry and the requirement for hospitalization. All enrolled patients were divided into four groups (stages 1–4) based on disease severity as recommended by the PAoEaI. Stage 1 included patients who were asymptomatic or mildly symptomatic, had oxygen saturation of at least 95% and did not require hospitalization due to COVID-19. Stage 2 included symptomatic patients with oxygen saturation lower than 95% who required hospital admission. Stage 3 included patients who started to develop respiratory failure and had oxygen saturation lower than 90%. Stage 4 included critically ill patients with developed acute respiratory distress syndrome (ARDS) who were in need of mechanical ventilation and ICU treatment.

### Ophthalmological questionnaire

An ophthalmologic questionnaire to assess the presence and severity of ocular symptoms and their duration at the time of examination in the emergency department of the hospital and in the previous preceding 7 days was performed. In addition, the survey took into account the patient's current ophthalmic disorder history, e.g., contact lens use, anterior eye injuries, past eye surgeries, allergic seasonal conjunctivitis and dry eye syndrome. Eye-COVID scores (ECSs) were as follows: 0 (absent) or 1 (present) for the following parameters: (i) swelling of the eyelids, (ii) itchy eyes, (iii) eye burning, (iv) excessive tearing, (v) redness of the eye, (vi) feeling of sand under the eyelids, (vii) presence of discharge, (viii) sticking of the eyelids, (ix) photophobia, (x) feeling of stiffness in the eyeball, (xi) eye pain, (xii) visual impairment, (xiii) cloudy vision, and (xiv) blurry vision.

### Swab collection and material analysis

Conjunctival and nasopharyngeal samples were obtained from all the subjects recruited for the study. Conjunctival swabs were collected after obtaining positive SARS-CoV-2 results from nose swab analysis. Material collections were performed from the lower conjunctival sac using swab sets and virus inactivating buffer R9F (A&A Biotechnology, Gdańsk, Poland). Viral RNA isolation was performed using the MagMAX™ Viral/Pathogen II Nucleic Acid Isolation Kit (Thermo Fisher, Waltham, MA, USA) according to the manufacturer’s protocol. Two hundred microliters of each sample was added to the designated sample well and mixed with 5 μL of proteinase K, 5 μL of MS2 phage control, 265 μL of binding buffer, and 10 μL of magnetic beads. Two hundred microliters of nuclease-free water was also pipetted into the negative control well in the sample plate. Furthermore, 3 processing plates (KingFisher™ 96 Deep-Well Plate) with Wash 1 Solution (500 µl per well), Wash 2 Solution—80% ethanol (1000 µl per well) and Elution Solution (50 µl per well) were also prepared. MagMAX Viral/Pathogen nucleic acid isolation was processed using an automated KingFisher™ Flex instrument (Thermo Fisher, ON, CA). RT–qPCR assays for detecting SARS-CoV-2 RNA were carried out using a QuantStudio™ 5 PCR instrument and a TaqPath™ COVID 19 CE IVD RT PCR Kit (Thermo Fisher) according to the manufacturer’s protocol. The one-step RT–qPCR contained 5 μl of RNA template/TaqPath™ COVID 19 Control, 6.25 μl of 4 × TaqPath™ 1 Step Multiplex Master Mix (Thermo Fisher, ON, CA), 1.25 µl of COVID-19 Real Time PCR Assay Multiplex and 7.5 µl of nuclease-free water in a total volume of 20 μl. The one-step RT–qPCR program included the RT reaction at 53 °C for 10 min, enzyme activation at 95 °C for 2 min and 40 cycles of PCR amplification at 95 °C for 3 s and 60 °C for 30 s. After RT–PCR was completed, the results were analyzed using Applied Biosystems™ COVID-19 Interpretive Software (Thermo Fisher): those tested were considered positive when at least 2 out of 3 analyzed SARS-CoV-2 genes (ORF1ab, N, S) had Ct values ≤ 37.

### Tear sample collection and analysis

Tears were collected at the time of admission to the hospital to assess the expression of proinflammatory cytokines in the tear film. Schirmer’s strips (TearFloTM, HUB Pharmaceuticals, Arizona, USA) were used to collect the tear fluid in all subjects. The strip was placed in the lower conjunctival sac, and tears were allowed to diffuse into the strip until reaching 20 mm on the strip scale. The subjects were allowed to blink freely during this time. The Schirmer strip was then placed into an Eppendorf tube and frozen to -80 °C. Tear fluid was extracted from Schirmer’s strips by agitating small cut pieces of these strips in 300 μl of phosphate buffered saline (PBS) solution in a sterile 1.5 ml microcentrifuge tube at 4 °C for 3 h. Tear fluid was then eluted by centrifugation for 10 min and stored at − 80 °C until further use. The concentrations of TNF-α, IL-1b, IL-2, IL-4, IL-5, IL-6, IL-8, IL-10, IL-12 p70, GM-CSF, and IFN-γ in tear fluids were measured by multiplex fluorescent bead-based immunoassays (Luminex Corporation, Austin, TX, USA) using commercial R&D Systems Luminex Performance Human High Sensitivity Cytokine Magnetic Panel A (R&D Systems, Minneapolis, MN, USA). Then, 100-µl aliquots of each standard, control and samples were added to the plate together with 25 µl of multiplex antibody capture microparticle solution, and the plate was incubated with agitation for 3 h. at room temperature. Subsequently, each well was washed with 100 µl of wash buffer 3 times using a hand-held magnet. Fifty microliters of detection antibody cocktail was pipetted into each well, and the plate was sealed and incubated at room temperature for 1 h on a shaker. After this step, the wash was repeated, and 50 µl of streptavidin–phycoerythrin mixture was added to the plate and incubated with agitation for 30 min in the dark. Finally, after washing, the microspheres in each well were resuspended in 100 µl of wash buffer and shaken at room temperature for 5 min. The plate was then read and analyzed on a Luminex 200 analyzer, and analyte concentrations were determined from five different standard curves showing MFI (median fluorescence intensity) vs. protein concentration.

### Statistical analysis

The statistical analysis was conducted using Statistica 13 software. The Mann–Whitney U test was used to compare quantitative and rank variables between groups. The strength of associations between quantitative and rank variables was measured with Spearman rank correlation coefficient (Rs). Fisher’s exact test was used to compare qualitative variables between groups. Quantitative variables were presented as mean ± standard deviation (SD) and/or median [interquartile range (IQR)]. A p value of less than 0.05 was considered significant.

### Institutional review board statement

The study was conducted according to the guidelines of the Declaration of Helsinki, and approved by the Bioethics Committee of the Pomeranian Medical University (protocol code KB-0012/83/2020, date of approval: 22.06.2020).

## Data Availability

The data used to support the findings of this study are available from the corresponding author upon request.
